# Environmental strigolactone drives early growth responses to neighboring plants and soil volume in pea

**DOI:** 10.1016/j.cub.2022.06.063

**Published:** 2022-08-22

**Authors:** Cara D. Wheeldon, Maxime Hamon-Josse, Hannah Lund, Kaori Yoneyama, Tom Bennett

**Affiliations:** 1 School of Biology, Faculty of Biological Sciences, University of Leeds, Leeds LS2 9JT, UK; 2 Graduate School of Agriculture, Ehime University, Matsuyama, Japan; 3 Japan Science and Technology, PRESTO, Kawaguchi, Japan

**Keywords:** strigolactones, neighbor detection, root exudates, plant-plant interactions, rhizosphere signaling, shoot growth

## Abstract

There has been a dramatic recent increase in the understanding of the mechanisms by which plants detect their neighbors,^1^ including by touch,^2^ reflected light,^3^ volatile organic chemicals, and root exudates.^4^^,^^5^ The importance of root exudates remains ill-defined because of confounding experimental variables^6^^,^^7^ and difficulties disentangling neighbor detection in shoot and roots.^8^, ^9^, ^10^ There is evidence that root exudates allow distinction between kin and non-kin neighbors,^11^, ^12^, ^13^ but identification of specific exudates that function in neighbor detection and/or kin recognition remain elusive.^1^ Strigolactones (SLs), which are exuded into the soil in significant quantities in flowering plants to promote recruitment of arbuscular mycorrhizal fungi (AMF),^14^ seem intuitive candidates to act as plant-plant signals, since they also act as hormones in plants,^15^, ^16^, ^17^ with dramatic effects on shoot growth^18^^,^^19^ and milder effects on root development.^20^ Here, using pea, we test whether SLs act as either cues or signals for neighbor detection. We show that peas detect neighbors early in the life cycle through their root systems, resulting in strong changes in shoot biomass and branching, and that this requires SL biosynthesis. We demonstrate that uptake and detection of SLs exuded by neighboring plants are needed for this early neighbor detection, and that plants that cannot exude SLs are outcompeted by neighboring plants and fail to adjust growth to their soil volume. We conclude that plants both exude SLs as signals to modulate neighbor growth and detect environmental SLs as a cue for neighbor presence; collectively, this allows plants to proactively adjust their shoot growth according to neighbor density.

## Highlights

• Pea plants detect neighbors through their root systems early in life
• Strigolactone exudation by neighbors is needed for this response
• Strigolactone exudation mutants are outcompeted by their neighbors
• Environmental strigolactones thus act as cue for neighbor presence

## Teaser

Wheeldon et al. show that pea plants change shoot growth in response to strigolactones exuded by neighboring plant roots. They show that plants that cannot exude strigolactones are outcompeted by neighbors and conclude that environmental strigolactones therefore act as a cue for neighbor presence in plants.

## Results and discussion

### Root-mediated responses to neighbors alter branching and biomass in pea

**Figure 1:**
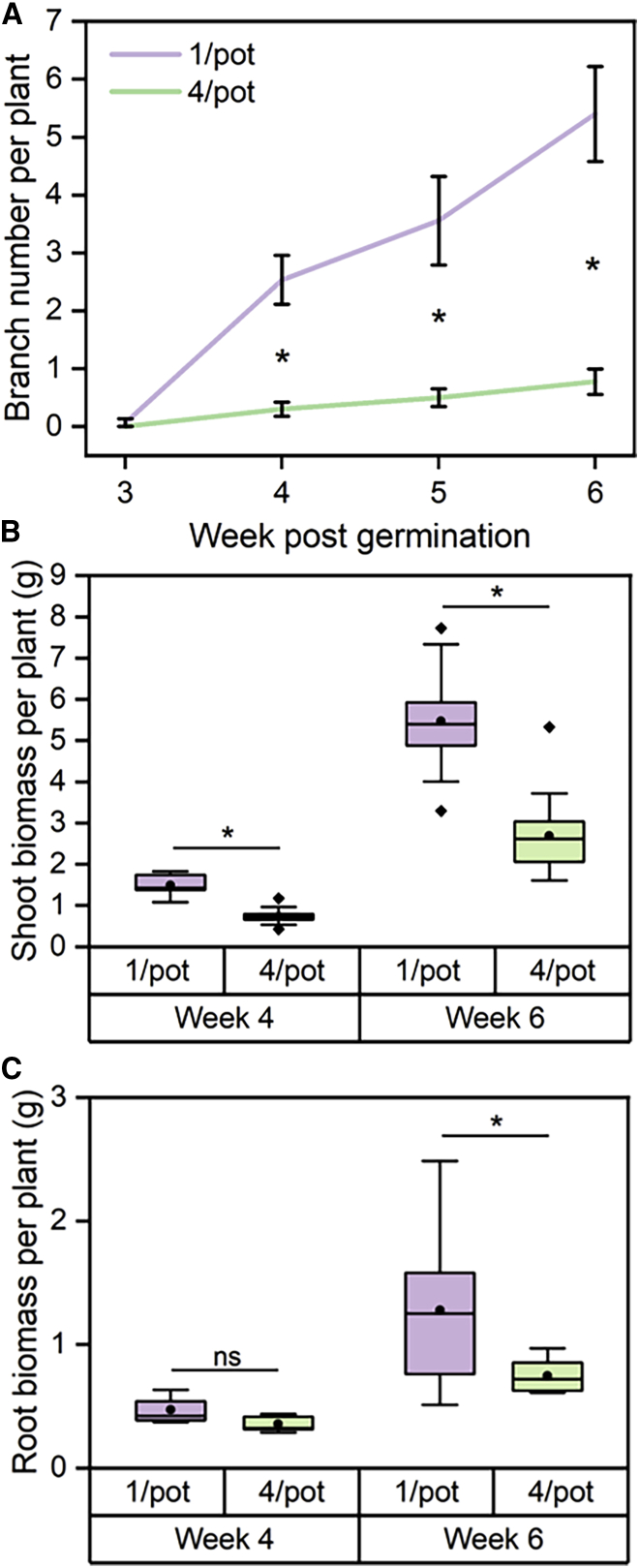
Response to neighbors occurs early in the pea life cycle (A) Primary branch number in WT pea plants over time. This was assessed weekly from 3 to 6 weeks post-germination. 1/pot, n = 15, 15, 9, and 10, respectively; 4/pot, n = 15, 15, 10, and 10, respectively. Error bars represent SEM. Asterisks indicate a significant difference between treatments (Mann-Whitney U test, p < 0.05). (B) Shoot biomass per plant (g) for each treatment at 4 and 6 weeks post-germination. 1/pot, n = 5 and 10, respectively; 4/pot, n = 5 and 10, respectively. Plants were destructively harvested, and their shoots were separated from the roots, dried for 3 days in a 60°C oven, and weighed. The box represents the interquartile range, whiskers represent the maximum and minimum values, the midline indicates the median, the • within the box represents the mean, and diamonds above and below whiskers represent outliers. Asterisks indicate a significant difference between treatments; 4 weeks, t test, p < 0.05; 6 weeks, Mann-Whitney U test, p < 0.05. (C) Root biomass per plant (g) for each treatment at weeks 4 and 6 post-germination. For measurements in week 4, each individual root system in the pot was separated from the shoot, dried, and measured before calculation of an average root biomass per plant within each pot. The data shown represent the distribution of these average values. 1/pot, n = 5; 4/pot, n = 10 pots. For measurements in week 6, 4/pot plant roots could not be separated, so these roots were dried and weighed together. The values presented represent the total root biomass in the pot divided by 4; the distribution of these values is shown. 1/pot, n = 5; 4/pot, n = 10 pots. The box represents the interquartile range, the whiskers represent the maximum and minimum values, the midline indicates the median, and the square within the box represents the mean. Asterisks indicate a significant difference between treatments (t test, p < 0.05); ns, no significant difference. See also Figure S1.

To assess whether strigolactones (SLs) play a role in neighbor detection, we first defined when pea plants respond to the presence of neighbors using a hydroponic system. We transferred 1-week-old wild-type (WT) pea plants to 1,000 mL hydroponic vessels, either singly (1/pot) or in square array of 4 plants (4/pot). Clearly, growing more plants in the same pot reduces the amount of nutrients available to each plant, which might influence the growth of the 4/pot plants. However, there is very good evidence that early responses to neighboring plants are driven by active signaling mechanisms, rather than long-term nutrient availability.^1^ Furthermore, in our hydroponic system, we were able to periodically replace the hydroponate to ensure that nutrient levels remained non-limiting; we are therefore confident that the responses observed in our experiments were not driven by nutrient limitation. We grew the plants for 5 more weeks, tracking the number of shoot branches, as well as destructively sampling some plants to measure shoot and root biomass 4 and 6 weeks into the experiment. We found that while the number of shoot branches was very similar at 3 weeks, from 4 to 6 weeks the 1/pot and 4/pot treatments strongly diverged in branch number (Figure 1A). Similarly, plants in the 1/pot and 4/pot treatments had very different shoot biomass after 4 weeks of the experiment (Figure 1B), although there was only a weak and statistically non-significant effect on root biomass at this time point (Figure 1C). All plants subsequently grew strongly between week 4 and week 6, with a ∼3.7-fold increase in shoot biomass in both 1/pot and 4/pot plants, thus precisely maintaining the differences present at week 4 (Figure 1B). Thus, the earliest neighbor-induced changes to shoot growth occur proactively, early in the life cycle, rather than in response to resource depletion later in the life cycle.

To determine whether the observed neighbor-detection effects were driven through shoot or root perception, we grew WT pea plants in either 500 or 2,000 mL soil, either 1/pot or 4/pot (Figure S1A). In 4/pot treatments, the plants were grown in the same proximity to each other irrespective of soil volume, and the shoots were staked so as to maintain this proximity throughout their height (Figure S1A). In this design, although shoot-mediated neighbor detection will necessarily increase between the non-crowded and crowded treatments, it should be constant between the different soil volumes *within* the crowded and non-crowded treatments. This design allowed us to quantify the general effects of crowding (same soil volume, different number of plants) and the specific effects of root system crowding (different soil volume, same number of plants). As anticipated from previous research, 4-fold crowding strongly inhibited shoot branching (by ∼3 fold) and shoot biomass (by ∼2.3 fold) in each plant in both soil volumes (Figures S1B, S1C, and S1E). As such, the total number of branches and the total biomass produced per pot are slightly larger in 4/pot treatments than in the equivalent 1/pot treatment (Figures S1B, S1D, and S1F). Conversely, increasing soil volume by 4-fold strongly stimulated production of shoot branches (by 2.6-fold) and shoot biomass (by 2.2-fold) (Figures S1B, S1C, and S1E), irrespective of crowding. When controlled for available soil volume (i.e., comparing 1/pot 500 mL and 4/pot 2,000 mL plants), the effect of crowding through the shoot system is a small 1.3-fold reduction in branching, consistent with the known mild effects of shading on branching.^21^ For shoot biomass, the effect of shoot system crowding was a negligible 1.09-fold reduction. Thus, the early neighbor-induced changes in shoot growth we observed are largely mediated through root-based detection.

### Early neighbor detection in pea requires SL biosynthesis

**Figure 2:**
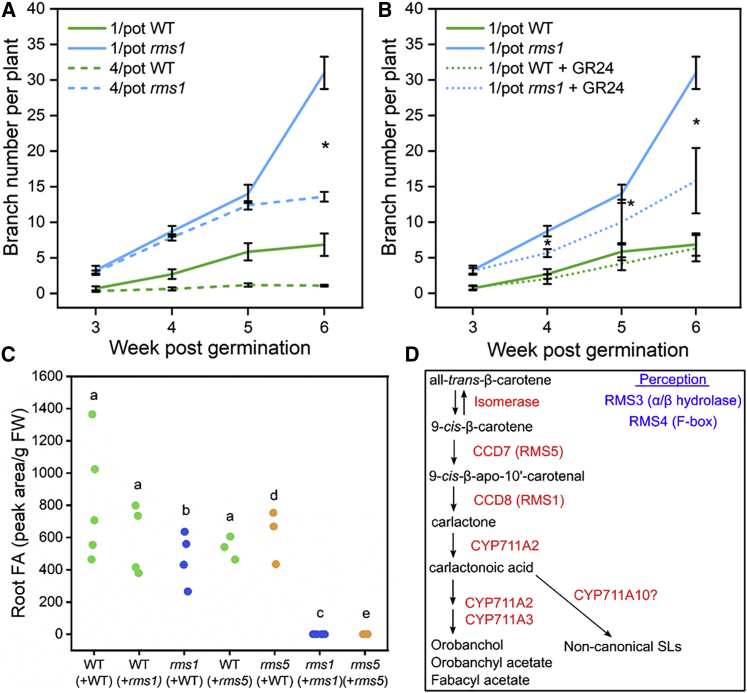
Strigolactone exudates can be taken up by neighboring plants (A) Line graph showing pea branch number per plant between weeks 3 and 6 post-germination. Solid lines represent 1/pot and dashed lines represent 4/pot for WT (green) and *rms1* (blue) plants. Average branch number per plant was calculated for each treatment for each week. 1/pot WT, n = 7, 7, 7, and 7; 1/pot *rms1*, n = 4, 4, 4, and 4; 4/pot WT, n = 6, 5, 5, and 5; 4/pot *rms1*, n = 6, 6, 6, and 6, respectively. Error bars represent SEM. Asterisks indicate significant difference between treatments for each genotype (t test with Bonferroni correction, p < 0.05). (B) Line graph showing pea branch number per plant between weeks 3 and 6 post-germination. Solid lines represent 1/pot WT (green) and *rms1* (blue) plants. Dotted lines represent 1/pot + GR24 for WT (green) and *rms1* (blue) plants. Average branch number per plant was calculated for each treatment for each week. 1/pot WT, n = 7, 7, 7, and 7; 1/pot *rms1*, n = 4, 4, 4, and 4; 1/pot WT *+* GR24, n = 6, 6, 6, and 6; 1/pot *rms1 +* GR24, n = 5, 5, 5, and 5, respectively. Error bars represent SEM. Asterisks indicate significant difference between treatments for each genotype (t test with Bonferroni correction, p < 0.05). (C) Two-plant hydroponic cultures of pea with 2× WT, 2× *rms1*, 2× *rms5*, 1× WT+1× *rms1*, or 1× WT+1× *rms5* were exposed to 7 days of P starvation. LC-MS quantification of FA was then performed on the root tissues of individual plants. x axis shows the tested plant and its partner (e.g., WT(+*rms1*) = FA levels in WT plants grown with *rms1* partner). Graph shows all individual data points; data expressed as peak area per gram fresh weight of root tissue. WT+WT, n = 5; *rms1*+*rms1*, n = 4; *rms5*+*rms5*, n = 3; WT+*rms1*, n = 4; WT+*rms5*, n = 3. Members of the same genotype with the same letter are not significantly different from each other (WT, ANOVA, Tukey-Kramer HSD test; *rms1*, t test; *rms5*, t test). (D) Diagram showing the SL biosynthesis pathway in pea, with chemical intermediates in black and enzymes (and gene names) in red. Genes required for SL perception are shown in blue text.

To test whether SLs act as root-emitted cues that mediate this early neighbor detection, we utilized *rms1* mutants, which do not synthesize SLs due to mutation of the CCD8 enzyme^19^ (Figure 2D). We used our hydroponic set-up, with either WT or *rms1-1* plants grown 1/pot or 4/pot. WT plants behaved as expected, strongly diverging in branch number between 3 and 4 weeks (Figure 2A). Due to the lack of SLs, *rms1* mutants have inherently higher branching levels than WT at all time points (Figure 2A). Unlike WT plants, we observed no divergence in shoot branching in *rms1-1* after 4 or 5 weeks. Thus, *rms1-1* plants appear unable to respond to the presence of neighboring plants early in development (Figure 2A). However, they were still ultimately able to detect and respond to the presence of neighboring plants, with shoot branching strongly accelerating in non-crowded *rms1* after 6 weeks, but not in crowded *rms1* plants (Figure 2A). This suggests there are two distinct stages in the shoot growth response to neighboring plants: a very early phase that requires SL and a second phase that does not.

### Strigolactone exudates can be taken up by neighboring plants

These data do not distinguish between two possibilities: that SL biosynthesis and exudation by neighboring plants are needed for focal plants to *detect* their neighbors (i.e., SLs are plant-plant signals) or that is only required in focal plants to *respond* to their neighbors (i.e., SLs are only involved in the downstream response to plant-to-plant signals). To distinguish between these possibilities, we first assessed whether plants are capable of taking up SL released by neighboring plants. There is certainly clear evidence that plants are highly sensitive to treatment with exogenous SLs,^15^^,^^16^^,^^18^^,^^22^, ^23^, ^24^ and in our experiments we found that application of 1 μM *rac*-GR24 to the roots of hydroponically grown *rms1-1* plants caused a significant reduction in branching (Figure 2B). To further demonstrate that pea plants can take up environmental SLs, we grew WT, *rms1-2T*, and *rms5-BL298* (lacking the CCD7 SL biosynthesis enzyme; Figure 2D) in co-culture of either the same genotype or in mixed genotypes, and then performed liquid chromatography-mass spectrometry (LC-MS) on the roots of individual plants to assess internal SL levels. We attempted to measure the concentrations of three pea SLs: fabacyl acetate (FA), orobanchol (OB), and orobanchyl acetate (OA). SLs are inherently low-abundance molecules that are difficult to precisely measure: we could only determine relative levels (“peak areas”) between samples rather than absolute concentrations; samples showed high variance; and in root tissues, only FA could be reliably detected. FA could not be detected in the roots of *rms1* or *rms5* plants grown in same-genotype co-culture, but was detected in the roots of both *rms1* and *rms5* plants that had been co-cultured with WT plants, with a corresponding reduction in FA levels in the corresponding WT plants relative to WT plants grown in same-plant co-culture (Figure 2C).

### Strigolactone exudation is required for early detection of neighboring plants

**Figure 3:**
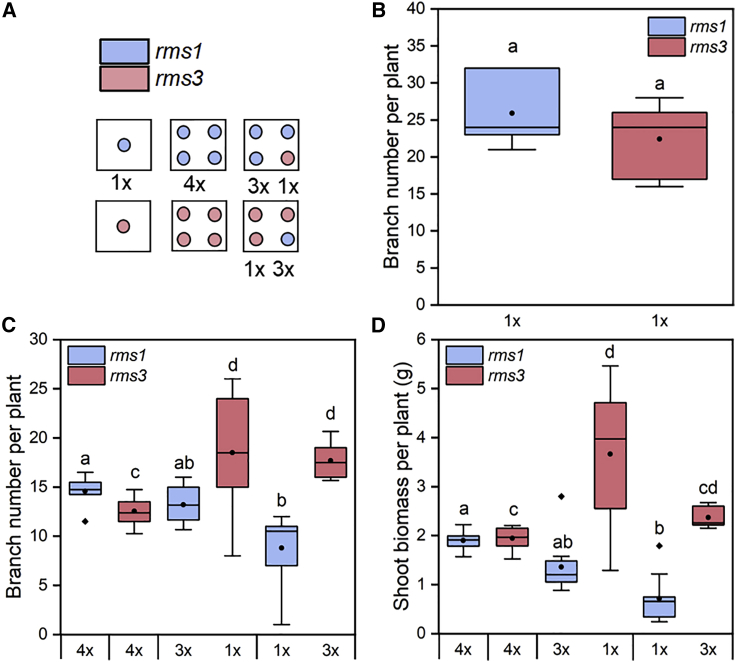
Strigolactone is required for early detection of neighboring plants (A) Diagram of the experimental set-up. Each blue dot represents an *rms1* plant and each pink dot represents an *rms3* plant. 1× represents 500 mL pots with one plant. 4× represents 500 mL pots with 4 plants of the same genotype. 3× + 1× represents 3 *rms1* plants and 1 *rms3* plant, respectively, in a 500 mL pot. 1× + 3× represents 1 *rms1* plant and 3 *rms3* plants, respectively, in 500 mL pots. (B) Boxplot showing branch number per plant 8 weeks post-germination for 1× *rms1* and 1× *rms3* pea plants. n = 10 for both *rms1* and *rms3*. The box represents the interquartile range, the whiskers represent the maximum and minimum values, the midline indicates the median, the • within the box represents the mean, and the diamonds above the box represent outliers. Boxes with the same letter are not statistically different from each other (Mann Whitney U test, p < 0.05). (C) Boxplot showing branch number per plant of *rms1* and *rms3* plants in crowding treatments 8 weeks post-germination. 4× *rms1*, n = 7; 4× *rms3*, n = 10; 3× *rms1*, n = 10; 1× *rms3*, n = 10; 1× *rms1*, n = 10; 3× *rms3*, n = 10. The box represents the interquartile range, the whiskers represent the maximum and minimum values, the midline indicates the median, the • within the box represents the mean, and the diamonds below the box represent outliers. Samples with the same letter are not statistically different from each other. Each genotype was only compared to itself among treatments (*rms1*, Kruskal-Wallis pairwise comparison, Bonferroni correction, p < 0.05; *rms3*, one-way ANOVA + Tukey HSD test, p < 0.05). (D) Boxplot showing dry shoot biomass (g) per plant of *rms1* and *rms3* plants in crowding treatments 8 weeks post-germination. Shoots were harvested individually, dried in a 60°C oven for 3 days, and then weighed. Individual shoot biomass was averaged across the treatment. 4× *rms1*, n = 7; 4× *rms3*, n = 10; 3× *rms1*, n = 10; 1× *rms3*, n = 10; 1× *rms1*, n = 10; 3× *rms3*, n = 10. The box represents the interquartile range, the whiskers represent the maximum and minimum values, the midline indicates the median, the • within the box represents the mean, and the diamonds above the box represent outliers. Samples with the same letter are not statistically different from each other. Each genotype was only compared to itself among treatments (Kruskal-Wallis pairwise comparison, Bonferroni correction, p < 0.05). See also Figure S2.

If SLs are plant-to-plant signals, then *rms1* should be “invisible” to its neighbors early in the life cycle since it does not synthesize SLs—but should still be sensitive to the presence of SL-exuding neighbors. Meanwhile, *rms3* mutants lacking a functional SL receptor (Figure 2D) should be insensitive to the presence of neighbors early in the life cycle. Conversely, if SLs are not plant-to-plant signals, but only required for the response to neighbors, *rms1* and *rms3* should be equally non-responsive to the presence of neighbors. Since our data indicate that SLs might indeed act as plant-to-plant signals, we hypothesized that if grown in co-culture, *rms1* plants would be unable to inhibit the early shoot growth of *rms3* plants but would be strongly inhibited themselves. An advantage of this co-culture system is that both plants have the same inherent shoot architecture, with short stems and high levels of shoot branching. Indeed, when grown 1/pot in 500 mL soil, the two mutants make a very similar number of shoot branches (Figure 3B). When grown 4/pot in same-genotype cultures in 500 mL soil (*4x rms1*; *4x rms3*), both mutants ultimately show reduced growth relative to 1/pot plants (consistent with the data shown in Figure 2A) and make similar numbers of shoot branches (Figure 3C) and similar shoot biomass (Figure 3D) to each other. However, when we grew 3 *rms1* plants with 1 *rms3* plant (*3x rms1 1x rms3*), we observed a highly significant increase in both the shoot branching and dry biomass of the solitary *rms3* plant (relative to *4x rms3*) and a small reduction in the shoot branching and dry biomass of the *rms1* plants (relative to *4x rms1*) (Figures 3C and 3D). This is consistent with our hypothesis: the three *rms1* plants now have one SL-exuding neighbor that inhibits their early shoot growth, and the solitary *rms3* plant cannot perceive any neighbors and grows correspondingly larger due to reduced competition. This trend is further amplified in *1x rms1 3x rms3*; we observed a significant reduction in the branching and biomass of the solitary *rms1* plant, together with a significant increase in the branching and biomass of all 3 *rms3* plants (Figures 3C and 3D). This is consistent with the solitary *rms1* plant having three SL-exuding neighbors that strongly inhibit its early shoot growth, which in turn allows the 3 *rms3* plants to grow larger relative to *4x rms3*. We also performed similar experiments with mixed cultures of WT and *rms1* (where shoot architecture is a potential confounding variable), with essentially the same results. WT plants cannot detect *rms1* and grow larger than expected in proportion to the number of *rms1* plants, while *rms1* plants can detect WT plants and grow smaller than expected in proportion to the number of WT plants (Figure S2). Overall, these data strongly support our hypothesis that SLs act as plant-to-plant signals, rather than simply in the response to plant-to-plant signals.

### Strigolactone exudation is homeostatically regulated in response to neighboring plants

**Figure 4:**
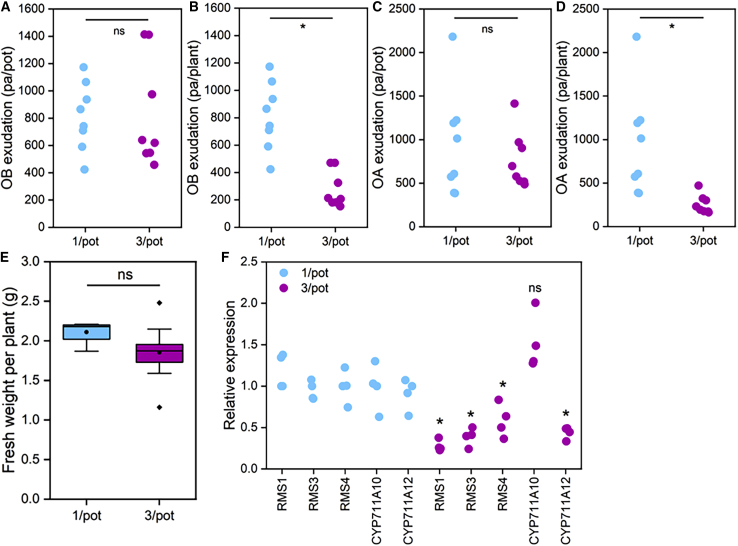
Strigolactone exudation is homeostatically regulated in response to neighboring plants (A–D) LC-MS quantification of OB (A and B) and OA (C and D) present in the hydroponate of 1 or 3 plant cultures of WT pea 5 days post-crowding, expressed as peak area (PA) per pot (A and C) or per plant (B and D). All data points shown. 1/pot, n = 4; 3/pot, n = 4. Asterisks indicate a significant difference from 1/pot; ns indicates no significant difference from 1/pot (A and B, Mann Whitney U test, p < 0.05; C and D, t test, p < 0.05). (E) Boxplots showing shoot and root fresh weight in grams shown as fresh weight per plant. The box represents the interquartile range, the whiskers represent the maximum and minimum values, the midline indicates the median, the • within the box represents the mean, and the diamonds above the box represent outliers. ns indicates no significant difference from 1/pot (Mann Whitney U test, p < 0.05); 1/pot, n = 4; 3/pot, n = 12. (F) Expression of *RMS1*, *RMS3*, *RMS5*, *CYP711A10*, and *CYP711A12* in root tissues of individual plants grown in 1/pot (blue) or 3/pot (magenta) plant cultures of WT pea after 12 days of P starvation. For all data points shown, 1/pot, n = 4 biological replicates; 3/pot, n = 4 biological replicates. Each data point is the average of 3 technical replicates. Expression levels for each gene normalized to 1/pot plant 1 (=1). Each genotype was only compared to itself among treatments. Asterisks indicate a significant difference in expression relative to 1/pot (t test, p < 0.05). See also Figure S3.

To further investigate the role of SLs as plant-to-plant signals, we measured environmental concentrations of FA, OB, and OA at various time points after pea plants were exposed to crowding (or left uncrowded) in a hydroponic system. FA is much more abundant than OA and OB, with OB sometimes falling below detection levels. Nevertheless, across 10 time points in 4 independent experiments, we observed a relatively consistent pattern in the data. For OB, the total concentration in the pot was essentially equal for the 1/pot and 3/pot treatments at all time points (Figures 4A and S3); as such, the exudation per plant was 3-fold lower in the 3/pot treatment (Figure 4B). For OA, a similar pattern was seen, albeit with more variability, particularly at later time points (Figures 4C and S3), while for FA, a different pattern was seen with significantly higher levels of FA in 1/pot treatments (Figure 4D), until the later time points, when there appeared to be some equilibration between the treatments (Figure S3). Importantly, at earlier time points these changes in SL biosynthesis occur without significant changes in plant size (Figure 4E). These data strongly suggest that downregulation of SL biosynthesis and exudation occurs in response to the presence of neighboring plants, an observation supported by the 2- to 3-fold reduction in the expression of the SL biosynthesis genes *RMS1*, *RMS5*, and *CYP711A12* (Figure 2D) in 3/pot plants relative to 1/pot plants (Figure 4F). Moreover, in the case of OA and OB, the downregulation seems to be homeostatic, with biosynthesis adjusted to maintain a consistent concentration of environmental OA/OB. Since homeostatic autoregulation of SL biosynthesis is well established,^25^ these data are consistent with exuded OB and OA acting as exuded signals that trigger downregulation of SL biosynthesis in neighboring plants. Consistent with this idea, there is no significant reduction in SL exudation by WT plants co-cultured with *rms1* plants compared to plants grown in isolation (Figure S3).

### Functional differentiation among strigolactones in rhizosphere and hormonal signaling

In order for plants to detect the presence of neighbors via OB/OA, there must initially be a higher concentration of OB/OA in crowded versus non-crowded plants before homeostatic reduction in SL biosynthesis occurs. There is some indication that this may be the case for OA at the earliest time points measured (Figure S3), but we were unable to reliably measure OB at those time points. However, in a companion manuscript, we show that environmental OB concentrations in rice match predictions for a plant-plant signal, being initially ∼3-fold higher in plants crowded by 3-fold, before homeostatic downregulation of SL biosynthesis leads to an equilibrium in environmental SL at around 72 h post-crowding.^26^ Thus, our data show that at a molecular level, the response to neighboring plants begins within a matter of days. Our data firmly suggest that FA is *not* a plant-plant signal since environmental FA levels are generally much lower in crowded than uncrowded plants (Figure S3C). Since FA is much higher in abundance than OB/OA, this could suggest that FA is exuded as a signal to AMF, while OB/OA are exuded as plant-plant signals. The well-known, but previously unexplained diversity of exuded SLs^27^ might thus reflect functional specification for different rhizospheric signaling roles.

Our data also pose an apparent paradox: root SL biosynthesis in crowded plants is reduced, but the inhibition of shoot branching and growth, a well-known effect of SLs, is increased. One obvious explanation for this might be that SLs are not the root-to-shoot signal that communicates neighbor detection; cytokinin is an obvious alternative.^28^ However, recent research suggests an emerging picture in which the canonical SLs do not act as hormonal signals within plants; this is the role of non-canonical SLs, which in turn do not act as exuded signals.^29^^,^^30^ Thus, although there is a reduction in the higher-abundance canonical SLs in crowded plants, this does not necessarily imply a reduction in the lower-abundance non-canonical SLs. In this context, it is notable that expression of *CYP711A10* is not decreased in crowded plants, unlike all other SL biosynthesis genes (Figure 4F). This suggests that there may be a “synthetic shunt” in crowded plants, with emphasis switching from canonical to non-canonical SLs amid a general reduction in SL biosynthesis, as plants seek to reduce both shoot growth and exudation of SLs.

### Understanding the benefits of environmental strigolactone exudation and perception

An intriguing question arising from our data is whether plants “eavesdrop” on SL “cues” released by neighbors as signals to AMF, or whether plants actively exude SLs as “signals” to control the growth of their neighbors. Our results suggest that SL exudation—in terms of short-term growth—primarily benefits the emitting plant, and not the responder (Figure 3). If this is the case, then why do plants remain sensitive to environmental SLs? Our recent work in wheat shows that plants emit and detect the concentration of a “substrate volume-sensing signal” (SVS), which allows them to detect their available soil volume and proactively modulate their shoot growth to match the long-term availability of soil resources.^29^ Like SLs in this study, an SVS primarily affects shoot growth, and its effects are first visible between 3 and 4 weeks post-gemination.^31^ As such we believe that SLs are SVSs. Consistent with this, we found that *rms1* mutants do not respond to soil volume over the first 5 weeks of growth, unlike WT plants (Figure S4). Thus, plants can clearly benefit in the long term by responding to the concentration of environmental SLs, by correctly adjusting their long-term growth to available resources. The same argument can be extended to neighbor detection; since neighboring plants invariably reduce the future availability of soil resources, detecting neighbor-exuded SLs helps plants shape their shoot growth to match long-term resource availability. The selective advantage of detecting environmental SLs—whether self- or non-self-exuded—is therefore the avoidance of resource limitations over the lifetime of the plant.

It is well established that many plants upregulate SL exudation in response to phosphate depletion in order to recruit AMF fungi.^32^ However, plants also exude considerable amounts of SLs in nutrient-sufficient conditions, which leave them prone to root parasitism by plants in the Orobanchaceae, which have evolved to detect SLs exuded by potential hosts.^33^ Our data suggest that SL exudation may be needed to maintain the competitiveness of plants among their neighbors, which makes constitutive exudation of SLs more comprehensible. Indeed, our data tentatively suggest that these two roles might be performed by different exuded SL species, with OB and OA acting in plant-to-plant signaling in pea, while FA is a highly inducible AMF signal. We are only just beginning to understand the meaning of the great diversity of SL isoforms, including the distinction between the signaling roles of canonical and non-canonical SLs; our data suggest further levels of complexity may exist within these classes as well. A role for SLs as plant-to-plant signals in *Physcomitrella patens* has previously been inferred, which could support an ancestral role of SLs as a plant-plant signal in land plants.^34^ However, there is no evidence for SL perception in liverworts or hornworts, though both taxa make SL and recruit AMF, abilities that have been lost in mosses.^35^ This perhaps makes it more likely that SLs have independently evolved as plant-plant signals in mosses and seed plants.

Consistent with our demonstration that environmental SL levels tend to achieve inter-plant homeostasis, the most stable evolutionary strategy may be for all plants to both “honestly” advertise their presence to their neighbors and “communally” respond to their neighbors by downregulating their growth and SL exudation. Overall, SLs are very likely only one component of root-based neighbor detection in flowering plants. Our data from *rms1* show that at least one other mechanism acts later in development to adjust shoot growth to the presence of neighbors. The timing of this effect is consistent with a second “root density-sensing” signaling system that wheat plants use to adjust their growth to their soil volume.^31^ Members of the Brassicaceae remain capable of detecting each other despite lacking significant SL exudation,^36^ and other exudates including jasmonic acid have been demonstrated to act in neighbor detection.^37^ While more work will be needed to understand the full complexities of underground plant-plant interactions, our data demonstrate the active nature of these processes and their importance in regulating plant growth.

## STAR★Methods

### Key resources table

**Table:** 

REAGENT or RESOURCE	SOURCE	IDENTIFIER
**Experimental models: Organisms/strains**		

*Pisum sativum* L77 Wild-type	Christine Beveridge^38^	N/A
*Pisum sativum* rms1-1 (L77 background)	Christine Beveridge^38^	N/A
*Pisum sativum* Torsdag Wild-type	Catherine Rameau^39^	N/A
*Pisum sativum* rms1-2T (Torsdag background)	Christine Beveridge^39^	N/A
*Pisum sativum* rms3-1 (Torsdag background)	Catherine Rameau^40^	N/A
*Pisum sativum* rms5-BL298 (Torsdag background)	Christine Beveridge^41^	N/A

**Oligonucleotides**		

RMS1-F (AAGGAGCTGTGCCCTCAGAA)	IDT	N/A
RMS1-R (ATTATGGAGATCACCACACACCATCA)	IDT	N/A
RMS3-F (TTGAGCAAGGGGAAATTGAG)	IDT	N/A
RMS3-R (TCTCTAACGGCTGTCGGAAC)	IDT	N/A
RMS5-F (CGGCATCTTAAAGACTCCGTACA)	IDT	N/A
RMS5-R (TGGATACGATCGGGAAGTTCA)	IDT	N/A
CYP711A10-F (TGTCTCTCCATTGGTTGCAAGA)	IDT	N/A
CYP711A10-R (CATACCCATGTTCCCTTTGG)	IDT	N/A
CYP711A12-F (CGTATCGCCATTAGTTGCAAGA)	IDT	N/A
CYP711A12-R (CAAACCCAAGTTCCCTTTGG)	IDT	N/A
Act-F (GTGTCTGGATTGGAGGATCAATC)	IDT	N/A
Act-R (GGCCACGCTCATCATATTCA)	IDT	N/A

**Software and algorithms**		

SPSS v28	IBM	https://www.ibm.com/uk-en/products/spss-statistics

### Resource availability

#### Lead contact

Further information and requests for resources and reagents should be directed to and will be fulfilled by the lead contact, Tom Bennett (t.a.bennett@leeds.ac.uk).

#### Materials availability

No new materials were generated in this study.

### Experimental model and subject details

#### Pisum sativum

##### Plant materials

*Pisum sativum* wildtypes Torsdag and L77 were used in this study, the *rms1-1* (L77), *rms1-2T* (Torsdag) and *rms3-1* (Torsdag) and *rms5-BL298* (Torsdag) mutants have previously been described.^38^, ^39^, ^40^, ^41^

##### Plant growth conditions

For phenotypic and physiological experiments, plants were grown under glasshouse conditions with a 16 hour day and 8 hour night regime at 22°C. LED lights with an average light intensity of ∼250 μmol/m^2^s^-1^ were used. Soil based experiments were grown on Petersfield No.2 compost. For SL quantification and gene expression tests, plants were grown on a plant cultivation shelf with a 14 hour day and 10 hour night regime at 23°C. LED lights with an average light intensity of ∼330 μmol/m^2^s^-1^ were used.

##### Hydroponic experiments

For phenotypic experiments, plants were grown for 1 week on perlite, and equal sized plants were selected, perlite was removed and plants were transferred into the hydroponic system. Plants were grown in 1l black plastic pots either 1 plant per pot (1/pot) or 4 plants per pot (4/pot). Plants were inserted into modified shortened falcon tube stubs with the bottoms removed leaving only the lid, screw top and ∼2 cm of tube. Roots were carefully passed through a hole made in the falcon tube lid, a foam bung was placed in the lid below the root/shoot junction to keep the plant in place, and the ∼2 cm tube modification was screwed back onto the lid and this as a whole was placed into a hole in the lid of the 1l pot. Air stones provided aeration and were connected with tubing to an aquatic pump (All Pond Solutions, AP-12-Kit pump). Each pot was filled with 1l water plus standard ATS nutrient solution.^42^ The water level was maintained daily and fresh ATS was provided at the midpoint of the experiment (15 ml of the stock solutions normally used to make 1l of ATS). For *rac*-GR24 treatments, rac-GR24 dissolved in acetone was added to the medium after 2 weeks of growth to a final concentration of 1μM, with an equivalent volume of solvent control added to non-treated plants. This treatment was repeated at 4 weeks (using the assumption that GR24 concentration had previously declined to 0 μM).

For SL quantification and gene expression tests, a similar set-up was used. Plants were germinated and grown for 1 week in vermiculite, and equal sized plants were selected, vermiculite was removed, and plants were transferred into the hydroponic system. Plants were grown in 500 ml plastic pots either 1/pot, 2/pot or 3/pot. Hydroponic media were replaced every other day. Strigolactones were extracted from root exudates and tissues as reported previously.^43^

### Method details

#### Phenotypic assessments

For branch counts, all branches longer than 10 mm on the plant were counted at the stated timepoint. For shoot and root biomass measurements, tissue was collected at the stated timepoint and dried for 3 days in an oven at 60°C before being measured on a balance.

#### Extraction of strigolactones

Strigolactones were extracted from root exudates and tissues as reported previously.^43^ Briefly, the root exudates released into media were collected and extracted with ethyl acetate. The ethyl acetate solutions were dried over anhydrous MgSO_4_ and concentrated *in vacuo*. For extracting strigolactones from root tissues, the harvested fresh root tissues (ca. 1 g) were washed, then soaked in ethyl acetate in the dark at 4°C for 2 days, and then filtrated, washed with 0.2 M K_2_HPO_4_, dried over anhydrous MgSO_4_, and concentrated *in vacuo*. These samples were kept at 4°C until analysis.

#### Mass spectrometry

Strigolactones were analyzed using the Acquity UPLC System (Waters) coupled to a Xevo TQD triple-quadrupole mass spectrometer (Waters MS Thechnologies) with electrospray (ESI) interface.^26^ HPLC separation was performed with an ODS column (2.1 x 50 mm, Waters) with a linear gradient of 35% methanol (0 min) to 95% methanol (15 min). The column oven temperature was maintained at 40°C. LC-MS/MS analysis (MRM, multiple reaction monitoring) of proton adduct ions was performed with a triple quadruple/linear ion trap instrument (Xevo TQD; Waters) with an electrospray source. MRM transitions for orobanchol and orobanchyl acetate eluting 4.8 min and 6.6 min, respectively were monitored for *m/z* 347.1/97 at a collision energy (CE) of 22 V and *m/z* 347.1/233 at CE of 10 V with a cone voltage of 30 V. The MRM transitions of *m/z* 405.3/97 at a CE of 20 V and *m/z* 405.3/203 at 15 V with a cone voltage of 30 V were used for the detection of fabacyl acetate eluting at 5.8 min. Data acquisition and analysis were performed using MASSLYNX 4.1 software (Waters). MRM chromatograms are shown in Figure S4.

#### qRT-PCR

Total RNA was extracted from the roots (>100 mg) of RNeay Plant Mini Kit (QIAGEN) according to manufacturer’s instructions and quantified with a spectrophotometer Nano Drop One C (Thermo Fisher Scientific, #ND-ONEC-W). 1 μg of total RNA was used to synthesize the cDNA with the PrimeScript RT Reagent Kit with gDNA eraser (Takara Bio, Japan). Real-time PCR was performed by ΔΔCT method on a StepOnePlus real-time PCR system (Thermo Fisher Scientific, #StepOnePlus-01) with THUNDERBIRD SYBR qPCR kit (Toyobo, Japan). The PCR program was as following: an initial DNA denaturation at 95°C for 20 s; 40 cycles including a denaturation step at 95°C for 3 s, an annealing step at 60°C for 30 s and an extension step at 95°C for 15 s; and a melting curve from 60°C to 95°C. Actin was selected as an internal reference gene in this study. The specific primers used for qRT-PCR are available in the key resources table.

### Quantification and statistical analysis

Statistical analyses were performed in SPSS v28 (IBM Software). The statistical test used, sample sizes, and p values for each experiment are stated in the figure legends. For sample size, n represents the number of pots in each treatment, not the number of plants. Where multiple plants shared the same pot, their values were averaged to find a value for each pot. Data was tested for normality to determine the statistical test most suitable for each experiment.

## Data Availability

• All data reported in the manuscript will be made accessible by the lead contact upon request. • This paper does not report original code. • Any additional information required to reanalyze the data reported in this paper is available from the lead contact upon request

## Supplemental information

Document S1. Figures S1–S4

Document S2. Article plus supplemental information
