# Supplementary material for: Environmental strigolactone drives early growth responses to neighboring plants and soil volume in pea
Source: Curr Biol. 2022 Aug 22;32(16):3593–3600.e3. doi: 10.1016/j.cub.2022.06.063 (PMC9616727; doi:10.1016/j.cub.2022.06.063)
Supplement: Document S1. Figures S1–S4 [file mmc1.pdf]

Current Biology, Volume *32*

Supplemental Information

Environmental strigolactone drives early growth responses to neighboring plants and soil volume in pea

Cara D. Wheeldon, Maxime Hamon-Josse, Hannah Lund, Kaori Yoneyama, and Tom Bennett

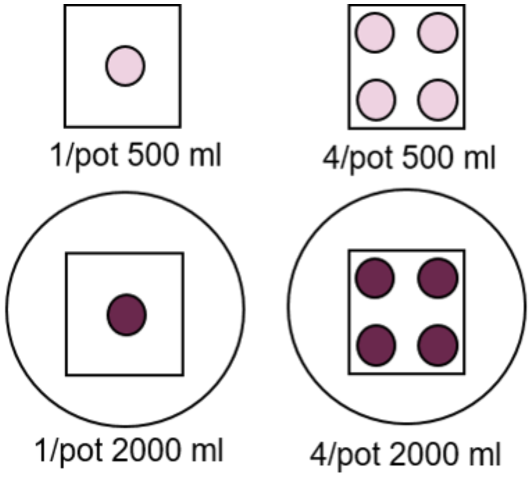

	500 ml → 2000 ml	1/pot → 4/pot
Branches per plant	2.6x (1/pot)<br>2.2x (4/pot)	3x (500 ml)<br>3.5x (2000 ml)
Biomass per plant	2.2x (1/pot)<br>2.1x (4/pot)	2.3x (500 ml)<br>2.4x (2000 ml)
Branches per pot		1.3x (500 ml)<br>1.1x (200 ml)
Biomass per pot		1.7x (500 ml)<br>1.7x (2000 ml)

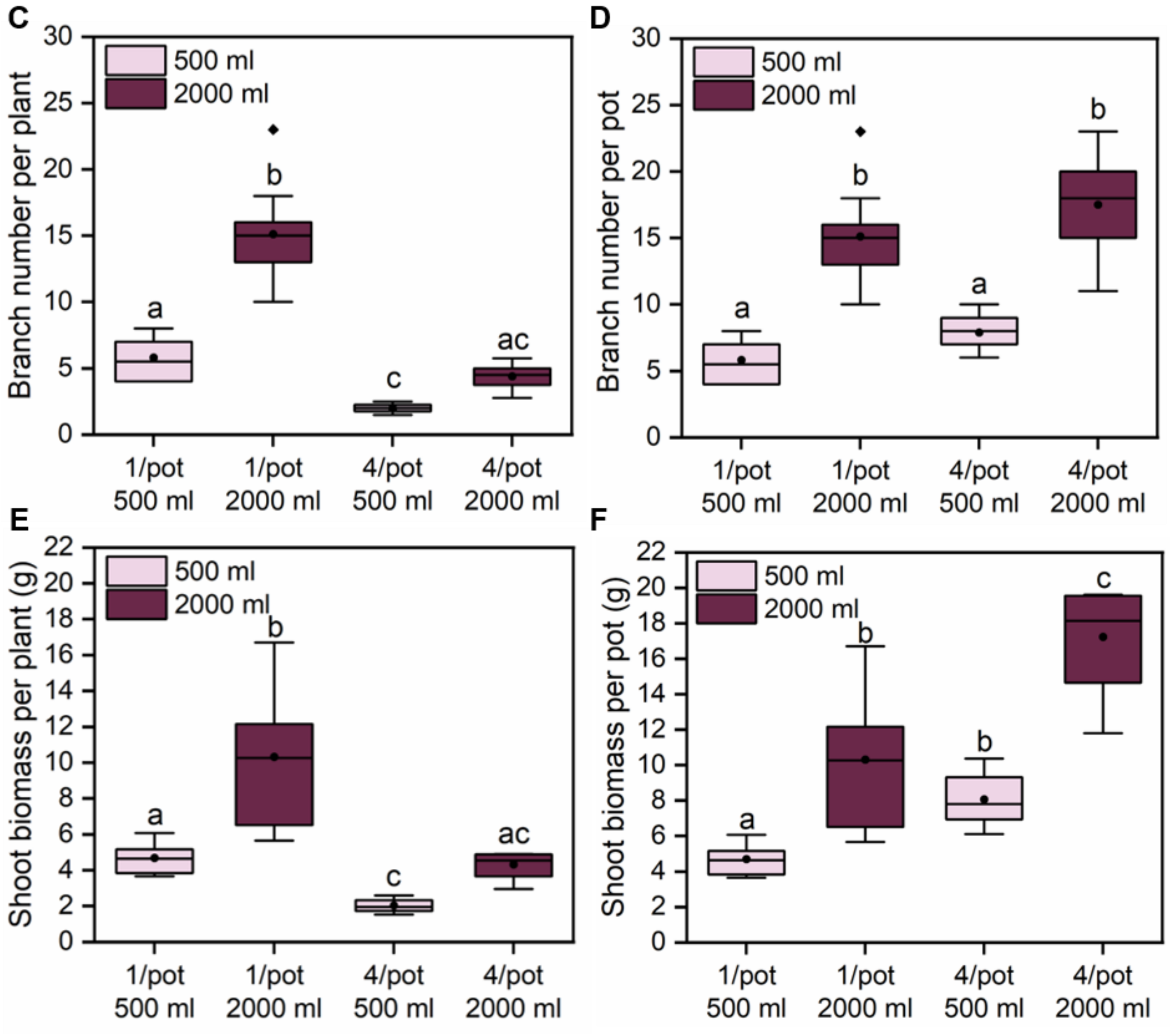

Figure S1: Root-mediated responses to crowding alter shoot branching and biomass in pea. Related to Figure 1

A) Cartoon representing the experimental set up. Plants were grown in either 500ml (pink) or 2000ml (burgundy) pots. The square within the 2000ml pots represents the same surface area as the plants sown in 500ml pots.

B) Table showing the fold change in branching and biomass per plant or per pot in response to increasing soil volume (500ml→2000ml) and crowding (1/pot→4/pot). Values in green are increased by the fold change, and those in red are reduced.

C) The number of branches produced 7 weeks post germination per WT Torsdag pea plant for each treatment. One plant per pot (1/pot) and 4 plants per pot (4/pot) were grown in 500ml (pink) and 2000ml (burgundy) pots. The box represents the interquartile range, whiskers represent the maximum and minimum values, the midline indicates the median, the • within the box represents the mean, diamonds above the box represent outliers. Branch number of each plant within a pot was averaged, from this an average was taken of these numbers across the pots in the treatment. n= 10, 10, 9, 10 pots per treatment, respectively. Bars with the same letter are not statistically different from each other (One-way ANOVA+ Tukey HSD test, $p<0.05$).

D) The number of branches produced 7 weeks post germination per pot of WT Torsdag pea plant for each treatment. One plant per pot (1/pot) and 4 plants per pot (4/pot) were grown in 500ml (pink) and 2000ml (burgundy) pots. The box represents the interquartile range, whiskers represent the maximum and minimum values, the midline indicates the median, the • within the box represents the mean, diamonds above the box represent outliers. The total number of branches produced per pot were averaged across the number of pots within the treatment. n= 10, 10, 9, 10 pots per treatment, respectively. Bars with the same letter are not statistically different from each other (One-way ANOVA+ Tukey HSD test, $p<0.05$).

E) The dry biomass per WT Torsdag pea plant for each treatment, 7 weeks post germination. The box represents the interquartile range, whiskers represent the maximum and minimum values, the midline indicates the median, the • within the box represents the mean. Dry biomass of each plant within a pot was averaged, from this an average was taken of these numbers across the pots in the treatment. n= 10, 10, 9, 10 pots per treatment, respectively. Bars with the same letter are not statistically different from each other (One-way ANOVA+ Tukey HSD test, $p<0.05$).

F) The dry biomass per pot of WT Torsdag pea for each treatment, 7 weeks post germination. The box represents the interquartile range, whiskers represent the maximum and minimum values, the midline indicates the median, the • within the box represents the mean. The dry biomass produced per pot was averaged across the number of pots within the treatment. n= 10, 10, 9, 10 pots per treatment, respectively. Bars with the same letter are not statistically different from each other (One-way ANOVA+ Tukey HSD test, $p<0.05$).

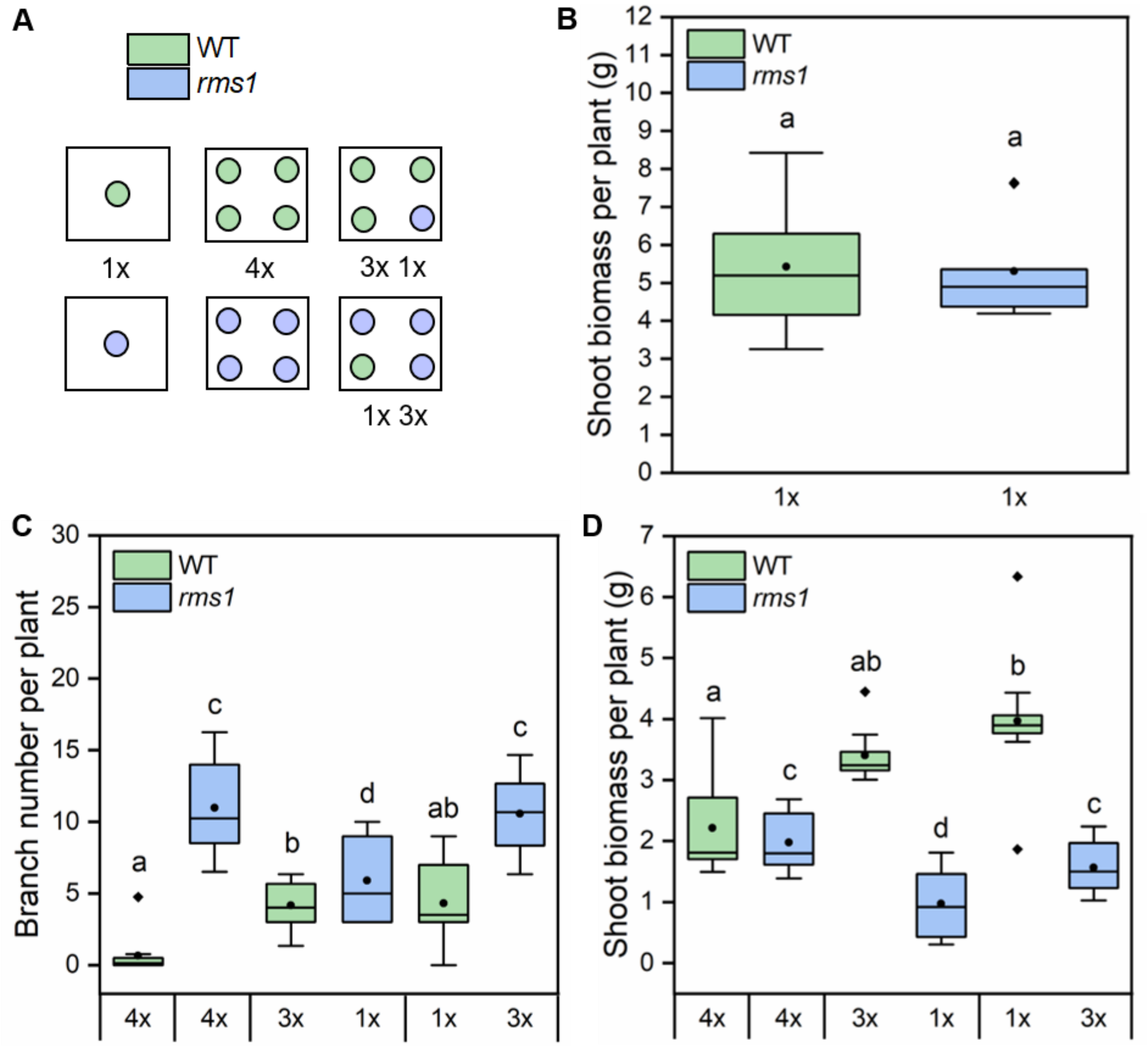

Figure S2: Strigolactone is required for early detection of neighbouring plants. Related to Figure 3

A) Cartoon of the experimental set up. A green dot represents 1 WT (L77) plant and a blue dot represents 1 *rms1* plant. 1x represents 500ml pots with one plant. 4x represents 500ml pots with 4 plants of the same genotype. 3x + 1x represents 3 WT plants and 1 *rms1* plant in a 500ml pot. 1x 3x represents 1 WT plant and 3 *rms1* plants in 500ml pots.

B) Box plot showing shoot biomass per plant of 1x WT and 1x *rms1* plants. n=10 biologically independent samples for all treatments. (The box represents the interquartile range, whiskers represent the maximum and minimum values, the midline indicates the median, the • within the box represents the mean,

diamonds below the box represent outliers. Boxes with the same letter are not statistically different from each other (t-test, $p<0.05$)

C) Box plot showing branch number per plant of WT and *rms1* plants in crowding treatments. n= 10 biologically independent samples for all treatments except 4x *rms1* where n=9. The box represents the interquartile range, whiskers represent the maximum and minimum values, the midline indicates the median, the • within the box represents the mean, diamonds below the box represent outliers. Boxes with the same letter are not statistically different from each other. Each genotype was only compared to itself among treatments (WT: Kruskal Wallis pairwise comparison, Bonferroni correction, $p<0.05$, *rms1:* One-way ANOVA+ Tukey HSD test, $p<0.05$).

D) Box plot showing shoot biomass (g) per plant of WT and *rms1* plants in crowding treatments. n= 10 biologically independent samples for all treatments. The box represents the interquartile range, whiskers represent the maximum and minimum values, the midline indicates the median, the square within the • represents the mean, diamonds above the box represent outliers. Samples of with the same letter are not statistically different from each other. Each genotype was only compared to itself among treatments (WT: Kruskal Wallis pairwise comparison, Bonferroni correction, $p<0.05$, *rms1:* One-way ANOVA+ Tukey HSD test, $p<0.05$).

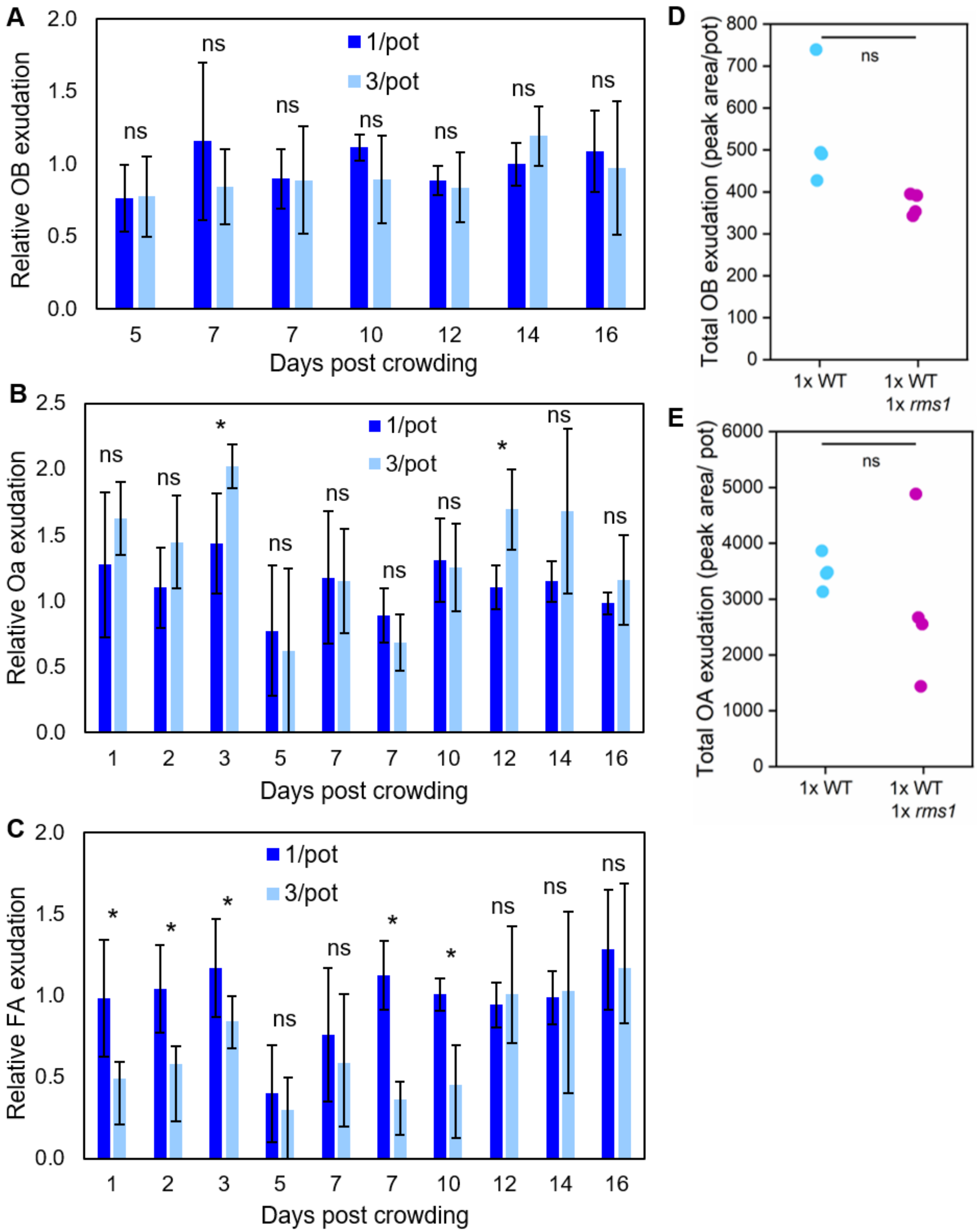
A
Relative OB exudation
2.0
1.5
1.0
0.5
0.0
1/pot
3/pot
ns
5
7
7
10
12
14
16
Days post crowding
B
Relative Oa exudation
2.5
2.0
1.5
1.0
0.5
0.0
1/pot
3/pot
ns
*
1
2
3
5
7
7
10
12
14
16
Days post crowding
C
Relative FA exudation
2.0
1.5
1.0
0.5
0.0
1/pot
3/pot
ns
*
1
2
3
5
7
7
10
12
14
16
Days post crowding
D
Total OB exudation (peak area/pot)
800
700
600
500
400
300
200
100
0
ns
1x WT
1x WT
1x rms1
E
Total OA exudation (peak area/ pot)
6000
5000
4000
3000
2000
1000
0
ns
1x WT
1x WT
1x rms1

Figure S3: Homeostatic regulation of environmental strigolactone levels. Related to Figure 4

A-C) Exudate levels of orobanchol (OB), orobanchyl acetate (OA) and fabacyl acetate (FA) in pots with 1 plant or 3 plants grown together (1/pot and 3/pot) measured by LC-MS across 10 time-points after crowding (including 7 days twice) from 4 separate experiments. Raw data were measured as peak area/pot, but to allow reasonable comparison between time-points, data here are shown as relative levels. The first measurement in each time-point was arbitrarily made equal to 1, and all other measurements in the same time-point were scaled relative to that measurement. Measurements within each time-point and treatment were then averaged, and standard deviations calculated, based on the relative data. These are shown in the bars and error bars, n=3-8 for each treatment, time-point. The test hypothesis is that there are no statistically significant differences between the 1/pot and 3/pot treatments, and application of the correct non-parametric tests with correction for multiple comparisons indeed shows no significant differences between any treatments at any time. However, to be maximally critical of the test hypothesis, the much more powerful student's t-test was used here, without correction for multiple comparison. Asterisks thus show where any possible significant differences in the data might occur (t-test, $p<0.05$, n=3-8), though for the reasons described above, these should not be taken as clear evidence for differences.

D,E) LC-MS quantification of orobanchol (OB)(D) and orobanchyl acetate (OA)(E) present in the hydroponate of 1-plant cultures of WT pea (A) or 2-plant cultures of 1 WT and 1 *rms1* pea plant, 14 days post-crowding, expressed as peak area (PA) per pot. All data points shown, n=4. ns indicates no significant difference from 1/pot (t-test, $p<0.05$).

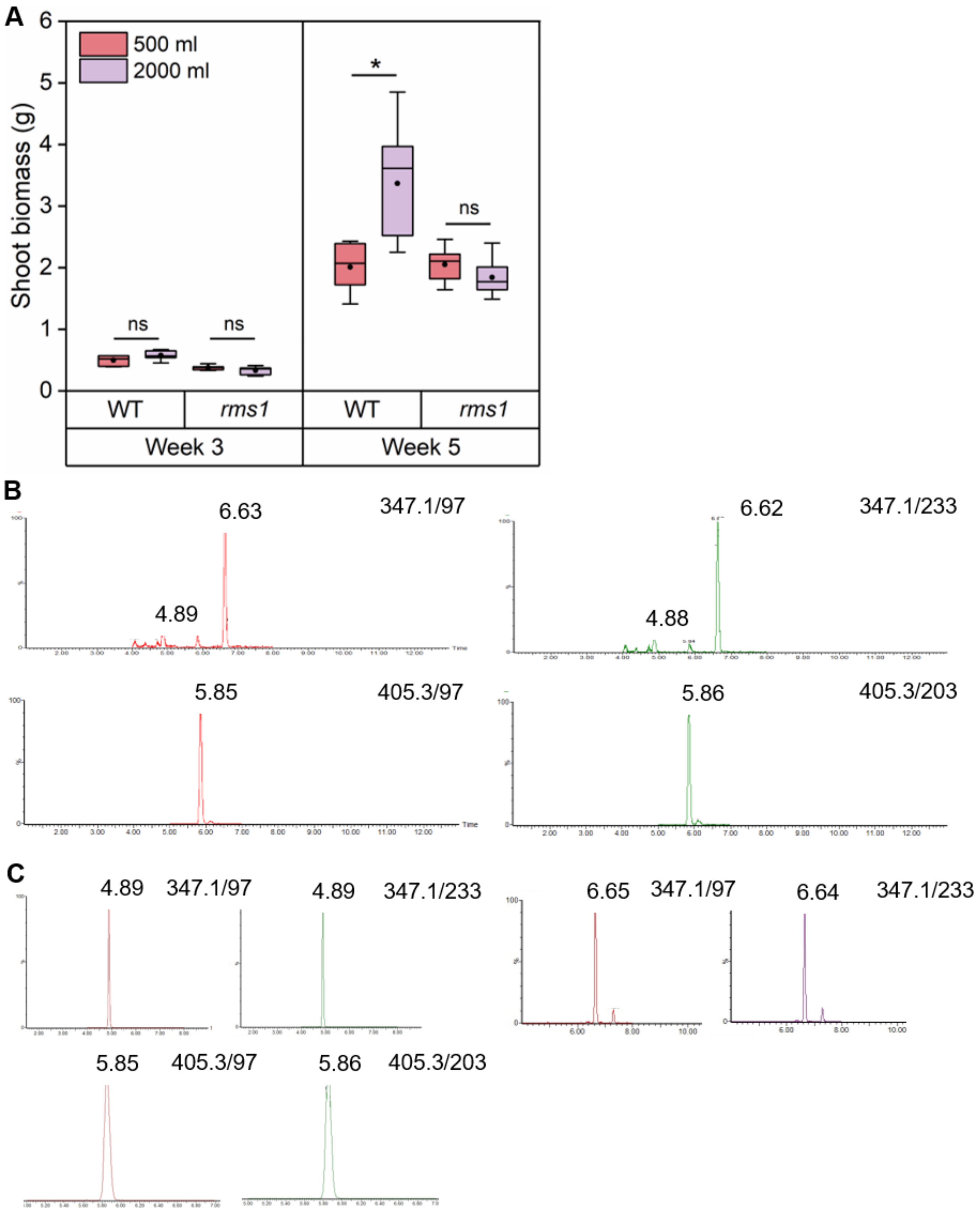
A
500 ml
2000 ml
Shoot biomass (g)
6
5
4
3
2
1
0
*
ns
ns
ns
WT
rms1
WT
rms1
Week 3
Week 5
B
6.63
347.1/97
4.89
6.62
347.1/233
4.88
5.85
405.3/97
5.86
405.3/203
C
4.89
347.1/97
4.89
347.1/233
6.65
347.1/97
6.64
347.1/233
5.85
405.3/97
5.86
405.3/203

Figure S4: Strigolactone exudation is required for early shoot growth responses to soil volume. Related to STAR Methods

A) Box plot showing dry shoot biomass per plant 3 weeks and 5 weeks post germination for singly grown WT and *rms1* plants in two soil volumes: 500ml (pink) and 2000ml (lilac). WT: n=6,6,10,10 and *rms1*: n= 6,6,10,10 respectively. The box represents the interquartile range, whiskers represent the maximum and minimum values, the midline indicates the median, the • within the box represents the mean. Boxes with the same letter are not statistically different from each other. Each genotype was only compared to itself at each time point. Ns indicates no significant difference, asterisks indicate a significant difference between the soil volume (T-tests, $p<0.05$).

B,C) Detection of orobanchol, orobanchyl acetate and fabacyl acetate in root exudates of pea (B) and those of authentic standards (C) by LC-MS/MS. MRM chromatograms of orobanchol, orobanchyl acetate (red, 347.1/97; green, 347.1/233; *m/z* in positive mode), and fabacyl acetate (red, 405.3/97; green, 405.3/203; *m/z* in positive mode) are shown. The peaks at 4.8, 6.6, and 5.8 min represent orobanchol, orobanchyl acetate and fabacyl acetate, respectively.
